# Supplementary material for: Collaborative intelligence in AI: Evaluating the performance of a council of AIs on the USMLE
Source: PLOS Digit Health. 2025 Oct 9;4(10):e0000787. doi: 10.1371/journal.pdig.0000787 (PMC12510544; doi:10.1371/journal.pdig.0000787)
Supplement: S2 File — (ZIP) [file pdig.0000787.s002.zip › S2 Data Raw data files containing unprocessed question inputs and LLM outputs including transcripts of deliberations/STEP3_Question-101-1_Type-MC-NJ.docx]

**Facilitator** **[Round 1]:** A 32-year-old woman comes to the office because of a 2-week history of burning rectal pain and bleeding. She says, ""The pain is like being burned with a hot poker."" The pain is at its worst when she has a bowel movement and for approximately 20 minutes after defecation. She rates the pain at its worst as an 8 on a 10-point scale. She has noticed small amounts of bright red blood on the toilet paper when wiping, as well as drops of blood on her underwear. Medical history is unremarkable and she takes no medications. BMI is 28 kg/m². Vital signs are normal. Abdominal examination discloses no abnormalities. The patient has pain during rectal examination. Anoscopy shows a small tear in the anal mucosa. Test of the stool for occult blood is positive. Which of the following is the most appropriate next step in management? (A) Colonoscopy (B) Excision and drainage (C) Internal banding (D) Oral docusate (E) Sclerotherapy

**AI_D:** RESTATING THE QUESTION: The question pertains to deciding the most suitable step in managing a 32-year-old woman with a two-week history of rectal pain and bleeding worsened by defecation, and a physical exam consistent with a small anal tear. The options proposed involve different therapeutic interventions including a colonoscopy (A), a surgical procedure (excision and drainage - B), a technique called internal banding (C), an oral stool softener (oral docusate - D), and a procedure known as sclerotherapy (E). PLANNING: The primary assumption should be that the patient's symptoms are being caused by the anal tear that was found during the anal exam, specifically known as an anal fissure. However, an alternative assumption could be that this may represent more serious pathologies like rectal cancer or inflammatory bowel disease. It's important to distinguish these possibilities because an anal fissure would likely respond to conservative management (like a stool softener), whereas more serious conditions would require more involved diagnostic and therapeutic interventions. CLINICAL REASONING: The clinical presentation of severe pain during and after bowel movements with bright red bleeding and a small tear in the anal mucosa noted on anoscopy is strongly indicative of an anal fissure. An anal fissure can cause significant pain due to the tearing of the sensitive anoderm, which can be exacerbated by bowel movements and could potentially lead to the presence of blood in the stool. Anal fissures are usually managed conservatively initially. Therefore, oral docusate (option D) would be a reasonable approach. This stool softener would help make bowel movements easier and less painful, promoting healing of the fissure. CONCLUDING: In contrast, colonoscopy, while vital for detecting colon cancer and other significant GI pathologies, might not be the first step in the management of a typical anal fissure as presented in this case. The options of internal banding, excision and drainage, and sclerotherapy, while useful in certain situations such as hemorrhoids and abscesses, would be too aggressive at this point for a common and typically benign condition like an anal fissure. RESPONSE: Therefore, the most appropriate next step in managing this patient's symptoms is oral docusate (Option D), in order to soften the stools and facilitate healing of the anal fissure indicated by the patient's symptoms and examination findings.

**AI_B:** RESTATING THE QUESTION: The patient is a 32-year-old woman presenting with severe rectal pain and bleeding which seems to be increased during and after bowel movements. She presents with a small tear in the anal mucosa revealed by anoscopy and positive occult blood test in stool. Given her symptoms and presentation, the best next step in management is sought. The options provided suggest various approaches. PLANNING: The assumptions from the information given are: (1) The patient might be experiencing an anal fissure, given the pain during and after defecation, the visible tear in the anal mucosa, and the positive occult blood test. (2) The primary issue might be the pain and bleeding as caused by the fissure. (3) The standard approaches to managing the condition range from conservative (Oral docusate - a stool softener) to invasive (Excision and drainage, Internal banding, Sclerotherapy). Also, performing a colonoscopy might be recommended under certain conditions. ALTERNATE ASSUMPTION: The patient's main issue could also be the consequential effects of the pain and bleeding from the fissure. Can these effects be mitigated before focusing on definitive treatment of the fissure? CLINICAL REASONING: In the context of anal fissures, pain and bleeding occur due to the rupture and irritation of sensitive anal mucosa, particularly during bowel movements. Conservative management typically includes stool softeners, such as Oral docusate, which can help reduce the trauma during defecation and promote healing of the fissure. Invasive procedures like excision and drainage, internal banding, or sclerotherapy might be reserved for cases where conservative management fails or is not feasible due to the severity or chronicity of the fissure. Similarly, a colonoscopy could be conducted if there's suspicion of other underlying gastrointestinal pathology, given the patient's age and relatively unremarkable medical history, this is a less likely cause of her presentation. CONCLUDING: Given the nature of symptoms, visible tear on anoscopy, and positive occult blood testing, this case likely points to an anal fissure. Considering that, the next best step should focus on pain relief and promoting healing by reducing strain during bowel movements. RESPONSE: Thus, option (D) Oral docusate appears to be the most appropriate next step in management. This is a conservative approach addressing the patient's main sources of discomfort and allowing the anal mucosal tear to heal naturally.

**AI_A:** (1) RESTATING THE QUESTION: The question is seeking to identify the most appropriate method of managing a 32-year-old lady presenting with symptomatic anal pain coupled with bleeding, which is at its peak during and shortly after bowel movement. The symptom description, examination findings, and investigations hint on anal fissure (a tear in the anal mucosa). This condition is characterized by pain and blood noticeable on wiping post-defecation. The patient's normal vital signs, abdominal examination and remarkable anoscopy results support this. (2) PLANNING: The potential assumptions here could be that the anal fissure is the result of constipation, obesity or other factors. A valid alternate assumption could be that it might be because of chronic strain or pressure on the anal canal, which could be linked to the patient's BMI of 28 kg/m². Thereby for managing, we could think about approaches that would relieve symptoms like pain and bleeding, while also addressing potential risk factors such as constipation, obesity, etc. (3) CLINICAL REASONING: If we connect these assumptions regarding the possible causes and the clinical manifestation of the anal fissure in this patient, we note that providing soft stools and avoiding strain during defecation could be beneficial. This will not only aid in pain management but also facilitate the healing of the mucosal tear. Though medical and surgical options are available, it is often preferred to start with medical management. This is because these patients often respond well to conservative management due to the nature of the acute anal fissures. (4) CONCLUDING: With this line of reasoning, an oral stool softener such as docusate is a viable option. Used primarily as a laxative, docusate reduces stool consistency, making it easier to pass. This results in less stretching of the anal canal and subsequent strain on the existing anal fissure during defecation. Therefore, docusate use is in line with the goal of effective management of the patient's pain and bleeding due to anal fissure, and is therefore clinically prioritized. (5) RESPONSE: Hence, the most appropriate next step in managing this patient should be 'Oral docusate' (Choice D). It will help alleviate the symptoms, enabling the anal mucosal tear to heal naturally without further strain or pressure.

**AI_C:** 1) RESTATING THE QUESTION: A 32-year-old woman presents with a 2-week history of severe burning rectal pain and bleeding. The pain is triggered by bowel movements and persists shortly after defecation, reaching intensity levels of 8 on a scale of 10. She also observes bright red blood on her underwear and toilet paper after wiping. Despite this, she remains in normal health and doesn't use medication. She's overweight with a BMI of 28 kg/m², but her vital signs appear normal. Physical examination does not disclose any abnormalities in her abdomen but provokes pain during the rectal examination. The anoscopy visualizes a small tear in the anal mucosa, and her stool examination returned positive results for hidden or 'occult' blood. The question then becomes, what is the ideal next step in her treatment given the choice between a colonoscopy, excision and drainage procedure, internal banding therapy, oral docusate treatment, and sclerotherapy? 2) PLANNING: In planning her treatment, let's consider the symptoms, which seem to indicate a common issue known as an anal fissure, some kind of small tear in the anal mucosa often associated with trauma like passing hard stools. Different treatments can be considered, like colonoscopy, to further investigate the gastrointestinal tract; excision and drainage, more suited for abscess; internal banding, more effective for hemorrhoids; oral docusate, a stool softener; or sclerotherapy, used more often for dilated blood vessels. 3) CLINICAL REASONING: The clinical presentation of rectal pain and bleeding associated with a small tear in the anal mucosa seen on anoscopy, points strongly toward an anal fissure. Colonoscopy might be warranted if we suspected broader concerns like colorectal cancer or other significant gastrointestinal pathology. However, given the patient's age and the specific set of symptoms focusing around the pain and blood associated with defecation, a broader systemic issue seems less likely. Excision and drainage would typically be used for an abscess, which does not fit this clinical history. Internal banding, a technique for removing hemorrhoids, wouldn't be suitable here, either as the issue focuses on a tear, not an outpouching. Lastly, sclerotherapy, which hardens and closes off problematic blood vessels, is not indicated here as there is a mechanical trauma (the tear), not a vascular issue causing the bleeding. 4) CONCLUDING: Given these assessments, the most appropriate next step would be to address the cause of the discomfort: the small tear. As hard stools often contribute to such tears, administering oral docusate can soften the stool and relieve the mechanical stress on the anal canal during bowel movements. The softer stool would allow the tear a better chance at healing and reducing incidents of blood presence. 5) RESPONSE: Therefore, the best option for management in this case would be (D) the administration of Oral docusate.

**AI_E:** RESTATING THE QUESTION: In other words, we are assessing a 32-year-old woman who has been experiencing a burning rectal pain, which worsens after defecation, and bleeding for the past two weeks. Her medical history shows no previous conditions, and she's overweight based on her BMI but otherwise, her vitals are normal. A physical examination was unremarkable except for severe discomfort during rectal examination. Anoscopically, a small tear in her anal mucosa was discovered, and her stool tested positive for occult blood. The question now is, what would be the most appropriate next course of action? PLANNING: Firstly, let's define a few terms: - Anoscopy is a procedure that uses an anoscope to see inside the anus and rectum. - Occult blood refers to the blood that is not visible without a microscope or chemical tests. - The options provided are a mix of diagnostic and treatment modalities: (A) Colonoscopy, a procedure used to see inside the colon and rectum. (B) Excision and drainage, typically used to treat an abscess. (C) Internal banding, essentially ligation of hemorrhoids. (D) Oral docusate, a stool softener. (E) Sclerotherapy, often used in vascular abnormalities. OBSERVATIONS: The lady has severe anal pain, especially after defecation, and there's noticed bleeding on her wiping tissue and underwear. The anoscopy revealed a 'tear' in the anal mucosa, which could be an anal fissure. This condition would explain the presentation of pain and bleeding after a bowel movement. CLINICAL REASONING: An anal fissure typically manifests as severe pain, often described as a cutting or tearing pain, during and after defecation, with fresh bleeding. Her presentation, along with the anoscopy findings consistent with an anal fissure, simplifies the diagnosis. It's also prominent that the pain has reached a point to which it's interfering with bowel movements, thus creating a vicious cycle where the fear of this intense pain may lead to withholding defecation, causing harder and larger stool, which in turn further traumatizes the anal canal and propagates the cycle. Therefore, alleviating this concern would be the primary target in management. Stool softeners like docusate help in such a scenario where (1) there's a need to prevent constipation, as strenuous efforts during defecation could exacerbate the tear and the cycle of pain, and (2) They ensure smooth bowel movements without traumatizing the anus, allowing the fissure to heal. CONCLUDING: Considering the nature of the clinical presentation, it's essential to break the ongoing cycle of retentive behavior due to anticipated pain, and soften the stool to allow passing without exacerbating the fissure. RESPONSE: Therefore, the administration of oral docusate (D) appears to be the most appropriate next step in the management of this probable anal fissure.

**Facilitator** The consensus among the AI responses is that the most appropriate next step in managing the 32-year-old woman's symptoms is the administration of oral docusate (Option D). The patient's symptoms of severe rectal pain and bleeding, which worsen during and after bowel movements, along with the physical examination findings of a small tear in the anal mucosa, strongly suggest an anal fissure. This condition is often caused by trauma such as passing hard stools, and can create a vicious cycle of pain and retentive behavior, leading to further trauma. Oral docusate, a stool softener, can help break this cycle by making bowel movements easier and less painful, thereby promoting healing of the fissure. It is a conservative approach that addresses the patient's main sources of discomfort and allows the anal mucosal tear to heal naturally. Other options such as colonoscopy, excision and drainage, internal banding, and sclerotherapy, while useful in certain situations, are not the first-line management for a typical anal fissure and may be too aggressive or not directly applicable to this case. Therefore, the consensus is that oral docusate is the most appropriate next step in managing this patient's symptoms.
